# Pre-COVID brain functional connectome features prospectively predict emergence of distress symptoms after onset of the COVID-19 pandemic

**DOI:** 10.1017/S0033291722002173

**Published:** 2022-07-26

**Authors:** Nanfang Pan, Kun Qin, Yifan Yu, Yajing Long, Xun Zhang, Min He, Xueling Suo, Shufang Zhang, John A. Sweeney, Song Wang, Qiyong Gong

**Affiliations:** 1 Huaxi MR Research Center (HMRRC), Department of Radiology, West China Hospital of Sichuan University, Chengdu, China; 2 Research Unit of Psychoradiology, Chinese Academy of Medical Sciences, Chengdu, China; 3 Functional & Molecular Imaging Key Laboratory of Sichuan Province, West China Hospital of Sichuan University, Chengdu, China; 4 Department of Psychiatry, University of Cincinnati, Cincinnati, Ohio, USA; 5 Department of Radiology, West China Xiamen Hospital of Sichuan University, Xiamen, 361000, China

**Keywords:** Brain connectome, COVID-19 pandemic, fMRI, depression, anxiety, posttraumatic stress, psychoradiology

## Abstract

**Background:**

Persistent psychological distress associated with the coronavirus disease 2019 (COVID-19) pandemic has been well documented. This study aimed to identify pre-COVID brain functional connectome that predicts pandemic-related distress symptoms among young adults.

**Methods:**

Baseline neuroimaging studies and assessment of general distress using the Depression, Anxiety and Stress Scale were performed with 100 healthy individuals prior to wide recognition of the health risks associated with the emergence of COVID-19. They were recontacted for the Impact of Event Scale-Revised and the Posttraumatic Stress Disorder Checklist in the period of community-level outbreaks, and for follow-up distress evaluation again 1 year later. We employed the network-based statistic approach to identify connectome that predicted the increase of distress based on 136-region-parcellation with assigned network membership. Predictive performance of connectome features and causal relations were examined by cross-validation and mediation analyses.

**Results:**

The connectome features that predicted emergence of distress after COVID contained 70 neural connections. Most within-network connections were located in the default mode network (DMN), and affective network-DMN and dorsal attention network-DMN links largely constituted between-network pairs. The hippocampus emerged as the most critical hub region. Predictive models of the connectome remained robust in cross-validation. Mediation analyses demonstrated that COVID-related posttraumatic stress partially explained the correlation of connectome to the development of general distress.

**Conclusions:**

Brain functional connectome may fingerprint individuals with vulnerability to psychological distress associated with the COVID pandemic. Individuals with brain neuromarkers may benefit from the corresponding interventions to reduce the risk or severity of distress related to fear of COVID-related challenges.

## Introduction

The worldwide spread of the coronavirus disease 2019 (COVID-19) pandemic has generated considerable fear and psychological distress since person-to-person communicability was established in late January 2020 (Holmes et al., 2020; Salari et al., 2020). With persistent waves of illness, there has yet to be a transition to normalcy and limited fear of transmission without a foreseeable endpoint (Ray et al., 2021). The pandemic has caused significantly increased burdens upon mental health care systems in addition to its well-known impact on physical health (Wang et al., 2020a; Zavlis et al., 2021). Given the pervasive life events encompassing unprecedented uncertainty of infection and enforced quarantine, related to the COVID-19 pandemic (Brooks et al., 2020), individuals are increasingly vulnerable to psychological distress problems, generally including depression, anxiety and stress (Prout et al., 2020; Shanahan et al., 2020). Notably, pandemic-related distress problems were more pronounced in adolescents and young adults than the elderly population (Huang & Zhao, 2020; Wang et al., 2020a), as they confronted increased media exposure to COVID-related information (Magson et al., 2021).

Furthermore, the individual psychological reactivity varies considerably when confronting multiple stressors (Liu et al., 2021), and the chronic stress conditions adherence to COVID-19 magnify the long-lasting ecological effects of the pandemic on populations even after the lockdown ended (He et al., 2021; Vindegaard & Benros, 2020). Individuals with proper coping strategies tend to have high resilience to protect themselves from severe distress (Kukihara, Yamawaki, Uchiyama, Arai, & Horikawa, 2014; Liu, Zhang, Wong, Hyun, & Hahm, 2020), while others may develop persistent distress symptoms due to chronic effects of the pandemic (Liu et al., 2021). Regarding great individual variations in psychological distress among general public during the pandemic (Zhang et al., 2020), exploring biomarkers to identify those who are at high risk for developing persistent distress symptoms may facilitate addressing the salient mental health issues (Chen et al., 2021). In addition, the exposure to COVID-19-related events and posttraumatic stress potentially exacerbate the psychological general distress during the pandemic (Liu et al., 2020), and it is of priority to unveil the relations of pandemic posttraumatic stress to the development of persistent general distress problems.

Brain functional connectome, representing a collective set of temporally correlated neural activation patterns, is rather customized among individuals as a neurobiological marker to fingerprint the individual differences toward psychological problems (Chahal, Kirshenbaum, Miller, Ho, & Gotlib, 2021; Rubinov & Sporns, 2010). In contrast to task-based manipulation with a specific task or stimuli, the functional connectome in resting-state may provide a more unbiased estimate for the persistent mental alterations (He et al., 2021). Prior studies revealed that intrinsic spontaneous activity and connectivity of brain default mode network (DMN) were the core neural mechanisms that how individuals respond to chronic stressors, and limbic and prefrontal substrates like the hippocampus and medial prefrontal cortex in the DMN served as the critical regions in the network resilience (Chang & Yu, 2019; Liu et al., 2021). In this case, functional abnormalities in the DMN were attributed to stress-related mental health issues such as general distress and posttraumatic stress (Joshi, Duval, Kubat, & Liberzon, 2020; van Ettinger-Veenstra et al., 2020). Despite extensive investigation toward underlying brain connectome of distress-related problems, the delineation of altered within- and between-network patterns during the pandemic from the perspective of macro-scale brain networks are poorly understood.

Herein, we explore the changes of psychological general distress between pre- and during-pandemic periods, and then build the altered brain-distress construct by characterizing the pre-COVID brain functional connectome that prospectively encodes distress alterations. We expected that the individual variations of brain connectome features prior to the publicized recognition of pandemic emergency state could identify those who are susceptible to continuous general distress, and components in the DMN dominated the connectome construction. Then we conducted the cross-validation analysis to further evaluate the predictive performance of the pre-COVID brain connectome. To uncover the indispensable role of the pandemic-induced posttraumatic impacts in the development of general distress, we investigate the underlying effects of the COVID-related posttraumatic stress on the brain-distress link via mediation analysis. We assumed that pandemic-induced posttraumatic stress may causally explain the relations of brain functional connectome to general distress alterations.

## Method and materials

### Data collection

**Fig. 1.**
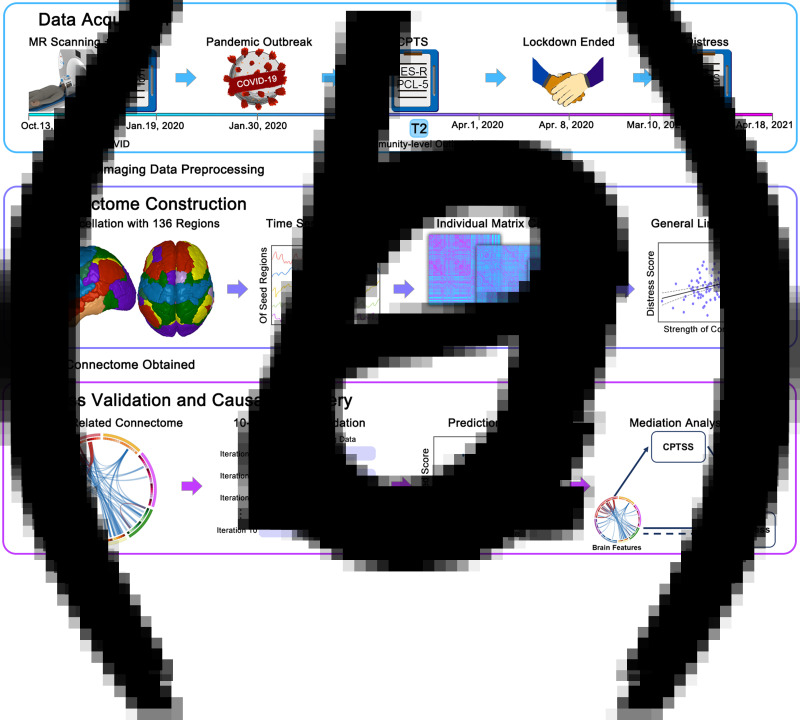
Schematic overview of the data acquisition and analytical procedures. Panel a: timeline of data acquisition and marked events of the COVID-19 pandemic in China. Notably, the World Health Organization declared the outbreak a Public Health Emergency of International Concern on 30 January 2020, and the Wuhan city lockdown officially ended on 8 April 2020 (indicating the remission of pandemic). Panel b: construction of distress-related functional connectome, from the brain region parcellation to prediction of distress with the general linear model. Panel c: cross-validation to examine the predictive performance of findings and mediation analysis to uncover the potential causal paths. DASS, Depression Anxiety Stress Scales; IES-R, Impact of Event Scale-Revised; PCL-5, Posttraumatic Stress Disorder Checklist for DSM-5; CPTS, COVID-19 posttraumatic stress, ΔDistress = pre-pandemic − during-pandemic distress score.

An overview of data collection and analytical procedures is shown in Fig. 1. A total of 151 individuals who had no known history of psychiatric or neurological disease were recruited for multimodal neuroimaging scanning and self-reported psychological scales from 13 October 2019 to 19 January 2020 (T1). Confirmation of human-to-human transmission of COVID-19 was established on 20 January and the declaration by the World Health Organization about the public health emergency of international concern occurred on 30 January (Mahase, 2020; Wang et al., 2020a). All participants were recontacted during the pandemic, and 127 participants responded and completed research procedures. Follow-up evaluations included COVID-related questionnaires to assess posttraumatic stress during the community-level outbreak and the peak from 1 February to 1 April 2020 (T2, the most severe pandemic period in China) and the same psychological scales with T1 from 10 March to 18 April 2021 (T3, post-peak period of the pandemic). Notably, these participants had not been infected with COVID-19 as established through nucleic acid testing. During these tests, 12 participants were excluded given that they failed to pass the bogus items that have only one correct answer (e.g. I was born on earth) to screen participants who are not paying attention and responding dishonestly (Desimone, Harms, & Desimone, 2015). Besides, 15 participants were excluded from further analyses due to excessive head motion (see Section ‘Neuroimaging data preprocessing’), and data from 100 participants (58 females, mean age 22.43 ± 2.12 years) were obtained for the primary analyses. This study was approved by the research ethics committee at the West China Hospital of Sichuan University. All individuals gave written informed consent before the participation.

#### MRI protocol procedure

Participants underwent magnetic resonance imaging (MRI) scanning using a Siemens-Trio Erlangen 3.0 Tesla system with a 12-channel head coil. Structural and resting-state functional MRI (rs-fMRI) data were obtained for each participant (details in online Supplementary methods).

#### Psychological general distress

To examine the general distress alterations between pre- and during-pandemic periods, participants were evaluated at T1 and T3 by the 21-item Depression Anxiety Stress Scale (DASS-21) (Henry & Crawford, 2005), which includes three subscales that could be generated into one factor – general distress (details in online Supplementary methods) (Zanon et al., 2021). To confirm that the previously reported one-factor structure of the DASS score could be used in our dataset, we performed the principal component analysis (PCA) in SPSS software (Version 24.0). We concluded that both waves of the DASS data corresponded to single-factor models (details in online Supplementary methods and Table S2). Thus, we used the total score of DASS as the measure of general distress symptoms, and distress alterations were calculated by subtracting the pre-pandemic and during-pandemic scores.

#### COVID-19 posttraumatic stress (CPTS)

To assess the impact caused by COVID-19 as a traumatic event on mental health problems, we employed the Impact of Event Scale-Revised (IES-R) (Wang et al., 2020a) and the Posttraumatic Stress Disorder Checklist for DSM-5 (PCL-5) at T2 (details in online Supplementary methods) (Blevins, Weathers, Davis, Witte, & Domino, 2015). The outbreak of the COVID-19 pandemic was specified as the stressor in the instructions of scales. Similarly, subscales of IES-R and PCL-5 (i.e. intrusion, avoidance, hyperarousal and cognition/mood) were dimensionally reduced into one factor by PCA (details in online Supplementary methods and Table S2). Therefore, the total score of IES-R and PCL-5 was computed and used as the measure of CPTS (Vindegaard & Benros, 2020).

#### Other measurements

Given that general stressful life events and socio-economic status may underlie the association between psychological distress, CPTS and brain functional connectome (Wang et al., 2020b), we also assessed the self-rating life events checklist (SLEC) (Liu et al., 1997) and socio-economic status scale (SESS) at the T1 to estimate their confounding effects (Peng et al., 2021). In addition, we asked participants whether their acquaintance or citizens in their communities were infected by COVID-19 at T3.

### Neuroimaging data preprocessing

The rs-fMRI data were preprocessed using GRETNA (https://www.nitrc.org/projects/gretna/) and SPM 12 (https://www.fil.ion.ucl.ac.uk/spm/) toolbox with the following steps: first 10 images remove, slice timing correction, realignment, coregistration of functional and structural images, normalization with DARTEL strategy, resampling to 3 mm^3^ isotropic voxels, spatial smoothing with a 6 mm full-width half-maximum, linear trend remove and temporal filtering at 0.01–0.08 Hz (Ashburner, 2007; Cui et al., 2020; Wang et al., 2015). White matter and cerebrospinal fluid signals and head motion parameters were regressed out as nuisance covariates. We calculated the mean framewise displacement (FD) of each participant and excluded those whose mean FD exceeded 0.25 mm, and we applied motion scrubbing based on the FD threshold of 0.50 mm for the remaining subjects (Yan et al., 2013). Finally, 15 participants were excluded for excessive head motion and 100 participants remained for subsequent analyses.

### Network matrix construction

To identify brain networks at the whole-brain level, the cerebral cortex was divided into 100 seed regions according to a 100-area parcellation proposed by Schaefer et al. (2018), and the subcortical seed regions were selected from 36 subregions in the human Brainnetome Atlas (Fan et al., 2016). Based on the neurobiological clustering of brain organization, the cortical brain areas were dimensionally reduced into Yeo's 7 preserved network structures, including the DMN, central executive network (CEN), ventral attention network (VAN), dorsal attention network (DAN), affective network (AFN), sensorimotor network (SMN) and visual network (VN) to enhance the interpretability of our findings without losing their granularity (Schaefer et al., 2018; Yeo et al., 2011). For subcortical regions, the striatal and thalamic parcels were grouped based on their preserved memberships with cortical networks (Choi, Yeo, & Buckner, 2012; Shirer, Ryali, Rykhlevskaia, Menon, & Greicius, 2012), and the subregions of amygdala and hippocampus were assembled with the AFN and DMN, respectively (Alves et al., 2019; Chin Fatt et al., 2020; Satpute & Lindquist, 2019). The brain seed region and network parcellation are shown in online Supplementary Fig. S1.

We obtained the average time series over all the voxels in each seed region for each participant. The Pearson's correlations between the time courses of all pairs of the regions were calculated using the GRETNA toolbox, and these correlation coefficients were transformed to *z* values for the normality. A 136 × 136 symmetric functional network matrix with 9180 links was constructed for each individual.

### Identification of functional connectome predicting distress

To identify the connectome patterns of the individual difference in distress alterations between the pre- and during-pandemic periods, we employed network-based statistic (NBS), which is a nonparametric statistical approach to control the family wise error (FWE) rate in multiple comparisons (Yang et al., 2019). The NBS based on permutation testing is a network graph analog of cluster-based thresholding of statistical parametric maps with promise for identifying distributed subnetworks cross massive links (Zalesky, Fornito, & Bullmore, 2010). The Pearson's correlation coefficients between each functional connectivity and the difference in distress were calculated in a general linear model with regressing out age, sex and mean FD. We collected the connections with the absolute *t* statistic exceeding an uncorrected threshold of 3.50 to detect interconnected subnetworks (Cocchi et al., 2012). The FWE-corrected *p* value was then assigned to each network with 10 000 permutations to yield a significant connectome. The corrected *p* value (*p* < 0.010) was computed when the recorded connectome was larger than or equal to the size of the network in the permuted data as the proportion of permutations (Cocchi et al., 2012). The identified connections of connectome were then assigned to within- and between-network relationships.

To recognize important brain regions (hubs) in the connectome, we used measures of node strength as the sum of connection strengths to a preserved seed region. Brain regions with a high node strength are likely to facilitate functionally interacting within connectome (Rubinov & Sporns, 2010).

### Individual prediction of distress alterations

To examine whether the identified pre-pandemic functional network component can robustly predict distress alterations at the individual level, we conducted a machine learning analysis with linear regression models (He et al., 2021; Lai, Wang, Zhao, Qiu, & Gong, 2020). The mean functional connectivity within the identified component was used as the feature to predict the individual differences in distress. To validate the robustness and generability of our prediction model, 10-fold cross-validation was implemented to train and test our model. Specifically, the whole dataset was randomly divided into 10 subsets, of which nine-fold were employed as the training data to build a linear regression model to predict the test data.

To evaluate the predictive capacity of brain connectome features on distress, we obtained the predicted values of difference in distress for the test data and computed *r*_(predicted, actual)_ to represent the correlations between the predicted and actual values (i.e. distress alterations) (He et al., 2021). The prediction framework was repeated 100 times to obtain the final *r*_(predicted, actual)_ to avoid the bias of train-test dataset split (Lai et al., 2020). Finally, we employed the permutation test that produced 5000 iterations to obtain a null distribution to assess the significance (Itahashi et al., 2020), and the *p* values were then determined as the percentage of permuted performance that was better than the original performance.

### Mediation analysis

We performed the mediation analysis using PROCESS macro in SPSS (Hayes & Scharkow, 2013) to investigate the role of CPTS in the predictive models of functional connectome to distress symptom changes. This approach has been widely used in studies to delineate a potential pathway linking brain features and behavior valuables via a mediator (Itahashi et al., 2020; Lai et al., 2020). The distress-related brain features (averaged *t* values of selected connections) were considered as the independent variables, the individual difference in distress alterations as the dependent variable, and the CPTS as the mediator. The direct and indirect effects of brain features on the difference in distress via CPTS were explored covarying age, sex and head motion. Based on bootstrapping approach (sampling = 5000), the indirect effect was significant when zero was not involved in the 95% confidence intervals (CIs).

## Results

### Pre- and during-pandemic psychological evaluations

The descriptive statistics and bivariate correlations for psychological assessments are shown in Table 1. According to the direction of distress change, 41 participants in the final sample were characterized as the increased distress group (mean age = 22.45, female proportion = 0.50, of which one subject reported people infected in their communities), and they exhibited significantly greater distress symptoms in the post-peak period of the COVID-19 pandemic compared to baseline (paired *t* test, *t*_40_ = 8.90, *p* < 0.001). Meanwhile, 54 participants (mean age = 22.48, female proportion = 0.63) showed decreased distress (paired *t* test, *t*_53_ = 8.66, *p* < 0.001, see online Supplementary Fig. S2). In addition, distress symptom alteration (T1–T3) was correlated with CPTS (*r* = −0.30, *p* = 0.002) but not with age (*r* = −0.09, *p* = 0.393), sex (*r* = 0.15, *p* = 0.142) or head motion (*r* = −0.12, *p* = 0.248). We then explored the relation of brain functional connectome to change of differences in distress symptoms from T1 to T3, and investigated the role of T2 CPTS in mediating this relationship.

**Table 1.** Psychological characteristics of sample

	Variables	Mean ± s.d.	Range	1	2	3	4	5
1	General distress (T1)	36.98 ± 9.39	21–57	–				
2	SLEC-frequency (T1)	12.73 ± 5.78	1–27	0.232*	–			
3	SLEC-impact (T1)	29.37 ± 16.95	2–77	0.272**	0.922**	–		
4	Family SES (T1)	9.88 ± 2.98	3–18	−0.404**	−0.226*	−0.227*	–	
5	CPTS (T2)	63.52 ± 21.00	42–151	0.255*	0.269**	0.357**	−0.086	–
6	General distress (T3)	35.74 ± 10.72	21–59	0.601**	0.236*	0.292**	−0.202*	0.481**

*Note*: The data were obtained from 100 participants (58 females, mean age = 22.43, age s.d. = 2.12). The distress symptoms were measured by the total score of Depression Anxiety Stress Scale, and the CPTS were assessed by that of Impact of Event Scale-Revised and Posttraumatic Stress Disorder Checklist for DSM-5. For detailed information of subscale, see online Supplementary Table S1. T1: October 2019 to January 2020 (pre-pandemic period); T2: February to April 2020 (community-level outbreak and peak of pandemic in China); T3: March to April 2021 (post-peak period). s.d., standard deviation; SLEC, Self-Rating Life Events Checklist; CPTS, COVID-19 posttraumatic stress symptoms; SES, Socio-economic status.

*p < 0.05, **p < 0.01.

### Brain connectome based on distress alterations

**Fig. 2.**
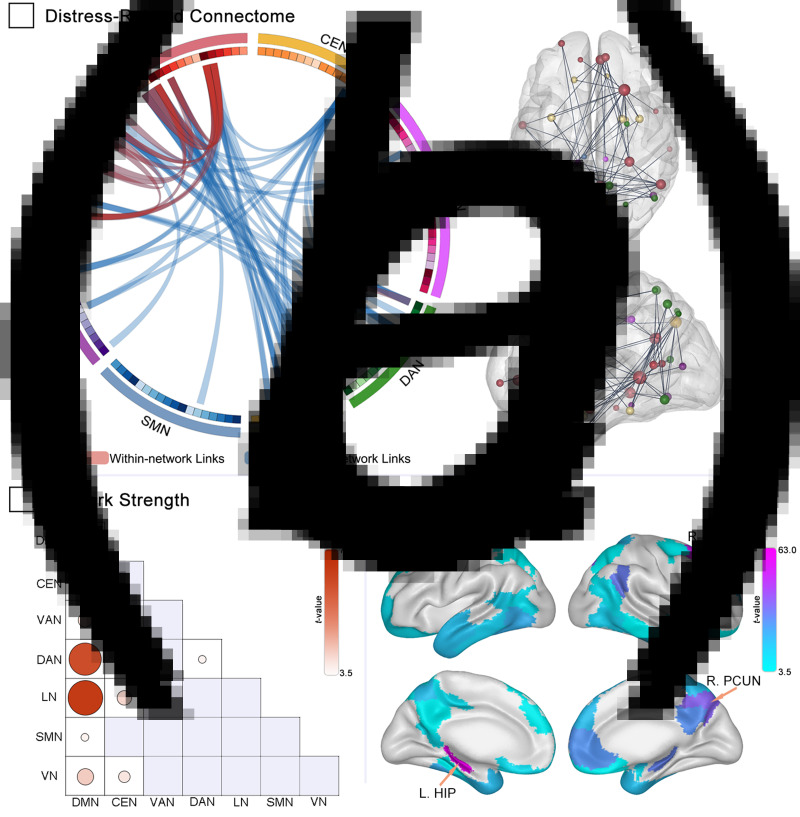
Functional connectome encoded difference in distress during the pandemic and the network and node strength of the connectome. Panel a shows the distress-related brain connectome with the connectogram in a circle plot, of which 136 brain regions are assigned to seven macroscale networks, and the connectome contains only 70 links over the threshold (details of links in online Supplementary Table S3). Panels b and c show the network and node strength that calculated by summing the correlation *t* value in specific network memberships and brain regions, respectively (details of brain regions in online Supplementary Table S4). DMN, default mode network; CEN, central executive network; DAN, dorsal attention network; AFN, affective network; VN, visual network; VAN, ventral attention network; HIP, hippocampus; dmPFC, dorsomedial prefrontal cortex; PCUN, precuneus.

The general linear model for network matrices identified brain functional connectome that prospectively encoded distress alterations with 70 negatively correlated connections after NBS-based correction for multiple comparisons (see Fig. 2*a* and online Supplementary Table S3 for identified connectome and online Supplementary Fig. S3 for connectivity matrix construct). The identified relevant connectome features allowed prediction of increased distress between pre- and during-pandemic periods (*r* ranged from 0.26 to 0.39, all *p* ⩽ 0.010), and weaker connected pair predicted severer distress symptoms after the pandemic outbreak.

Among these identified links, 20 links were assigned to within-network pairs (in a total of within-network 1422 links) and 50 links to between-network pairs (in a total of between-network 7758 links). Notably, most of the within-network connections located in the DMN (19/20 links, network strength = 77.44), and the links between the DMN and AFN (17/50 links, network strength = 63.45) and links between the DMN and DAN (15/50 links, network strength = 55.78) constituted the largest proportion of between-network pairs (Fig. 2*b*).

The node strength analysis revealed that the left caudal hippocampus (node strength = 63.08, DMN), right dorsomedial prefrontal cortex (node strength = 56.26, DMN) and right precuneus (node strength = 37.76, CEN) are the top three provincial hubs with more than 10 connections (Fig. 2*c* and online Supplementary Table S4), which may play a critical role in connectome resilience and robustness.

### Cross-validation of distress-related brain features

**Fig. 3.**
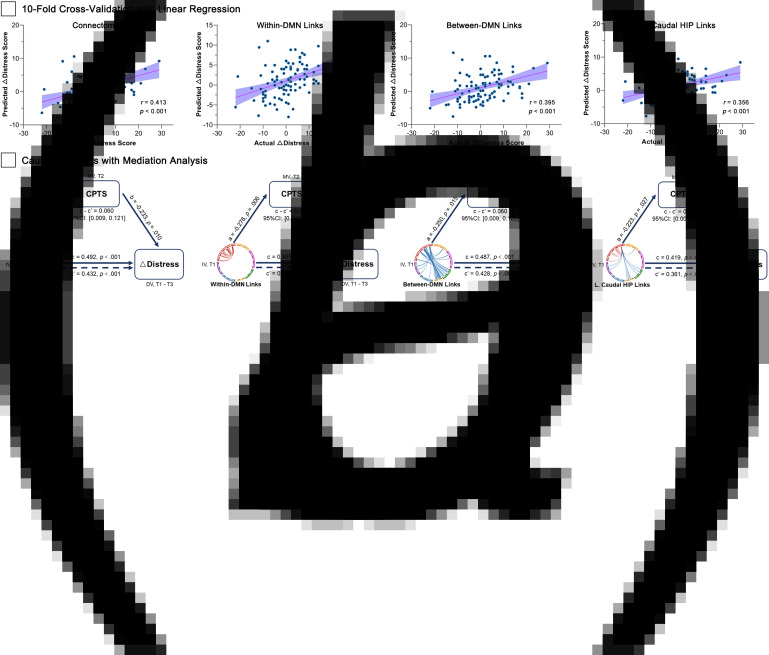
Prediction models based on various brain features and causal relations between variables. Panel a: prediction models for distress alterations exhibiting associations between the actual and predicted scores by 10-fold cross-validation with linear regression. Panel b: COVID-19 posttraumatic stress (CPTS) underlies the correlates between brain features and distress alterations. The indirect effect of CPTS (*c*–*c*′) is significant among the four models. Age, sex and head motion were regarded as covariates in the mediation analyses, and the coefficients in pathways (*a*, *b*, *c* and *c*′) were exhibited as standard regression coefficients. DMN, default mode network; L., left; HIP, hippocampus, ΔDistress = pre-pandemic − during-pandemic distress score.

Analysis of 10-fold cross-validation with linear regression suggested that the prediction of distress via functional connectome was found to be reliable (*r*_(predicted, actual)_ = 0.41, *p* < 0.001, Fig. 3). The distress alterations could also be predicted by the within-DMN links (*r*_(predicted, actual)_ = 0.42, *p* < 0.001), between-DMN links (*r*_(predicted, actual)_ = 0.40, *p* < 0.001), and left caudal hippocampal links (*r*_(predicted, actual)_ = 0.36, *p* < 0.001), respectively. Given the highly significant correlation and robust predictive performance, these brain features were employed as independent variables in the subsequent mediation analyses.

### Mediator role of pandemic-induced posttraumatic stress

After obtaining the brain functional connectome predicting general distress changes during the COVID-19 pandemic, we further investigated whether the CPTS could explain the brain-distress relations when adjusting for age, sex and head motion. We first examined that CPTS could be predicted by the whole connectome (*r* = −0.26, *p* = 0.011), within-DMN links (*r* = −0.28, *p* = 0.006), between-DMN links (*r* = −0.25, *p* = 0.015) and left caudal hippocampal links (*r* = −0.22, *p* = 0.027).

The findings so far indicated that brain connectome features, CPTS and general distress symptoms interrelated with one another, but the essence of the relations between them remained uncertain. To examine whether CPTS mediated the linkage of connectome features to general distress symptoms, we performed mediation analyses with age, sex and head motion as controlling variables. The results demonstrated that the CPTS partially explained the connectome-distress linkage (standard indirect effect = 0.06; 95% CI 0.01–0.12, *p* < 0.05. The mediation model held when selecting within-DMN, between-DMN and left caudal hippocampal links as independent variables (Fig. 3). In summary, the brain connectome features may affect general distress symptom alterations via the CPTS.

### Specificity of findings

To confirm the specificity of our main findings, we interrogated our results when controlling for scores of the SLEC and SESS as additional confounding factors. The supplemental analyses suggested that the association between the CPTS and distress alterations was also significant (*r* = −0.35, *p* = 0.001). Besides, the distress symptom change was significantly correlated with those brain connections (*r* ranged from 0.31 to 0.41, all *p* ⩽ 0.002). The mediation models persisted in the supplemental analysis as well (details in online Supplementary Fig. S4).

## Discussion

In a prospective investigation of public mental health following the unprecedented COVID-19 pandemic, we assessed the psychological states of a general population sample in the pre-pandemic, outbreak and post-peak periods. Brain features prior to COVID-19 epidemic could predict later emergence of distress, mainly including within- and between-DMN connection patterns with the left hippocampus emerging as a hub region. Analyses confirmed that posttraumatic stress induced by the COVID-19 pandemic played a mediator role in the brain-distress prediction. Our results were independent of other general stressful life events and family socio-economic status, indicating the specificity of the findings. Although a population-level increase in psychological distress may not be the case during the COVID-19 pandemic (Breslau et al., 2021; Daly & Robinson, 2021), additional efforts on quantitative data targeted to those at high risk for developing serious distress symptoms during the pandemic are warranted apart from merely baseline level of distress and resilience (Kimhi, Marciano, Eshel, & Adini, 2020; Shanahan et al., 2020). Neuroimaging data on volumetric alterations have been proved to be closely associated with anxiety and stress during the pandemic (Salomon et al., 2021). Furthermore, based on the brain connectome in fMRI modality, we may identify individuals who confer high vulnerability to long-standing distress symptoms with well-performing predictive modeling (Li, Sun, Biswal, Sweeney, and Gong, 2021). In this case, our findings may reveal neural correlates that prospectively encode pandemic-related distress at the individual macro-scale network level, and the susceptibility markers may serve as targets for early psychological and/or brain intervention that help to embark on long-term coping strategies in terms of limited mental health care resources (Chahal et al., 2021; He et al., 2021).

### Connectome in the network level

The identified connection patterns in the current study were predominantly associated with the DMN network, which was consistent with previous researches on neural correlates of psychological general distress. Our findings suggested that the weaker within- and between-network connections of DMN predicted more severe distress symptoms during the pandemic, indicating that the habitual functioning of DMN reversed the transition toward pathologically negative emotion as a resistant role (Akiki, Averill, & Abdallah, 2017; Chin Fatt et al., 2020; Qin et al., 2022). In view of neuroimaging, the DMN is characterized by resting-state brain functional activity during a cognitive task, by which individuals tend to experience mind-wandering that evokes negative emotions including depression and anxiety (Gusnard, Raichle, & Raichle, 2001; Mason et al., 2007; Zhang et al., 2022). The DMN is also involved in affective empathy (Göttlich, Ye, Rodriguez-Fornells, Münte, & Krämer, 2017), which indicates the simulation of others' emotional experiences that resulted in empathic distress (Ashar, Andrews-Hanna, Dimidjian, & Wager, 2017). The execution of DMN during rest allows to construct discrete emotions of negative valence by conceptualizing affective sensations in the context of preceded experience (Pan et al., 2022; Satpute & Lindquist, 2019), and the deprivation of temporal coherence in DMN may impair the processing of generalizing across heterogeneous emotion features (Lindquist, Wager, Kober, Bliss-Moreau, & Barrett, 2012; Suo et al., 2022), all of which may further lead to distress symptoms.

Specially, our findings of provincial within-DMN connectivity in the connectome demonstrated that the disruption of intrinsic synchronous coordination of DMN may be the predictors of subsequent pandemic-related distress, which is aligned with previous research studies that indicated its critical role as predictive biomarkers in individuals with major depressive disorder and posttraumatic stress disorder (Korgaonkar, Goldstein-Piekarski, Fornito, & Williams, 2020; Patriat, Birn, Keding, & Herringa, 2016). In addition, lower connectivity of the DMN with AFN and DAN in our identified connectome predicted more severe long-standing distress symptoms during the pandemic. The AFN located in the temporo-amygdala-orbitofrontal circuit is anchored on the interrogation of emotion with the encoding and retrieval of declarative memory (Pan et al., 2021; Petrides, 2007). Deficits in the memory framework resulting from weaker connections to AFN may thwart access to positive emotions and contribute to the psychopathology of distress, which formed negative behavioral strategies (Catani, Dell'Acqua, & Thiebaut de Schotten, 2013; Dobbin & Ross, 2019). In addition, the DAN is engaged in mediating and interpreting external stimuli in emotion regulation (Korgaonkar et al., 2020), and a previous study suggested that stronger connectivity between DMN and DAN was paralleled with contextualizing emotional experience during reappraisal (Froeliger et al., 2012; Sripada et al., 2014), with which individuals might overcome pandemic stressors at a mitigated distress level. Taken together, individuals with weak connections of DMN are more likely to indulge in internalizing processes and psychological distress triggered by predisposing scenes when exposing to information and life events adherence to the COVID-19 pandemic (Li et al., 2020; Satpute & Lindquist, 2019).

### Hub regions of the connectome

When considering local substrates of the connectome, the left caudal hippocampus, right dorsomedial prefrontal cortex and right precuneus appeared as hub regions in the connectome encoding distress alterations during the pandemic. Specially, the hippocampus, belonging to the medial temporal lobe subsystem in DMN, represents the psychological formation of scenarios in terms of autobiographical memory recollection (Akiki et al., 2017; Zhou et al., 2020). Individuals with posttraumatic stress disorder are characterized by the lower activity and weaker intrinsic connectivity of hippocampus (Akiki et al., 2017; Joshi et al., 2020). Similarly, growing literature of the COVID-19 pandemic also confirmed that pre-pandemic functional connectivity of hippocampus contributed to robust predictive markers of during-pandemic distress (Feng et al., 2021; Perica, Ravindranath, Calabro, Foran, & Luna, 2021). In view of psychophysiology, the hippocampus engages in glucocorticoid feedback inhibition that responds to external stressors in emotional information processing with a high density of hormone receptors (Chang & Yu, 2019). The temporal disconnection between the hippocampus and other substrates may underlie the suppressed synaptic strength that contributed to increased glucocorticoid levels and individual vulnerability to the long-standing exposure to threatening pandemic stimuli (McEwen & Gianaros, 2011). Furthermore, the dorsomedial prefrontal cortex subsystem of DMN is instrumental in mentalizing and processing current psychological states, and its hyper-activation is aligned with rumination as a core symptom of major depressive disorder (Satpute & Lindquist, 2019). Structural abnormalities of the dorsomedial prefrontal cortex were associated with the individual difference in psychological distress (Luo et al., 2018; Lai et al., 2022). Additionally, the activity of precuneus is anchored on highly integrated tasks including memory retrieval and self-referential processing (Cavanna & Trimble, 2006). The psychopathological form of self-processing is rumination that emerged as obsessively distressful thinking based on retrievals from personalized memory framework (Zhou et al., 2020), which might be the preceded symptom for developing pandemic-related distress.

### Mediation effects of posttraumatic stress symptoms

Our findings of mediation analysis revealed that COVID-related posttraumatic stress could independently explain the correlates of brain connectome to general distress alterations as a prominent resource. Previous studies suggested that severe posttraumatic stress symptoms were predicted by decreased intrinsic connectivity of DMN with abnormality in the hippocampus (Joshi et al., 2020; Patriat et al., 2016), underlying psychopathological processes of disrupted mentation, altered self-processing and fear generalization (Akiki et al., 2017). Regarding the role of DMN and hippocampus in emotion processing and affective empathy (Göttlich et al., 2017; Satpute & Lindquist, 2019), the weaker connectivity assigned to DMN regions may facilitate pointing out individuals who are highly susceptible to pandemic-related posttraumatic stress, which further contributes to persistent general distress symptoms.

### Confounding effects of sex

Our study did not find any correlations between distress symptom alteration and sex ratio. However, previous studies indicated that sex differences may play a critical role in the mental health crisis of pandemic, as females were more susceptible to affective hyper-reactivity (Perica et al., 2021; Shanahan et al., 2020). The discrepancy might be attributed to the limited sample size only when considering behavioral data. Given that early onset of puberty in females caused by many sociobiological factors may pose a threat to psychological distress (Natsuaki et al., 2009; Perica et al., 2021), the female population deserves more attention in terms of coping with this thorny issue.

### Limitations

The current study has several limitations that should be considered. First, our data were collected from uninfected young adults with COVID-19, of which findings may not generalize to other populations, and mental tolerance and resilience of general subjects may protect them from extremely high levels of distress symptoms during the pandemic (Liu et al., 2020). Further studies evaluating the predictive performance of functional connectome in the other general public populations (e.g. children and the elderly) and highly vulnerable groups (e.g. frontline medical workers) to psychological distress are warranted (Lin et al., 2020). Second, our predictive model only revealed individual varying degrees of distress alterations based on brain constructs when the distress scale was considered a dimensional rather than a psychopathological measurement (Henry & Crawford, 2005). Further study with a case-control design based on DSM-5 criteria might be needed. Third, we only collected the neuroimaging data before the epidemic outbreak without a follow-up longitudinal brain data to illustrate associations of pandemic event-related impact with brain connectivity, and our study design was unlikely to assess the robustness and consistency of identified brain-distress constructs across multiple time points (Weissman et al., 2021). Due to the exploratory nature of our study, yet the likelihood of this approach being used in clinical settings is limited. In terms of these limitations mentioned above, when coupled with perspective psychological data during the COVID-19 pandemic and preceded neuroimaging data in our study, longitudinally multimodal research focusing on highly vulnerable groups to mental distress might be of great priority to cope with the global health crisis.

## Conclusions

Psychological distress problems have captured widespread attention during the COVID-19 pandemic, especially for vulnerable young adults who are sensitive to stressors of pandemic-related events. In this regard, we identified a preceding brain functional connectome principally constituting DMN-connectivity patterns that encodes distress symptom changes based on a prospective psychological investigation among a young adult sample. The well-performing predictive models may interrogate individuals to determine if they are at high risk for developing pandemic-related persistent distress (Chahal et al., 2021). According to the functioning of DMN, those who are marked with the altered susceptibility connectome may benefit from mental construction of scenarios in psychotherapies (e.g. mindfulness-based training program; Zhou et al., 2020) and neural interventions (e.g. neurofeedback procedures; Sitaram et al., 2017) to reduce the risk of or mitigate distress symptoms adherence to COVID-related challenges, which is in line with the aim of psychoradiology (Gong, 2020; Lui, Zhou, Sweeney, & Gong, 2016).

## Supplementary material

For supplementary material accompanying this paper visit https://doi.org/10.1017/S0033291722002173. click here to view supplementary material
