# Supplementary material for: Pre-COVID brain functional connectome features prospectively predict emergence of distress symptoms after onset of the COVID-19 pandemic
Source: Psychol Med. 2022 Jul 26;53(11):5155–66. doi: 10.1017/S0033291722002173 (PMC9433719; doi:10.1017/S0033291722002173)
Supplement: Supplementary file 1 [file S0033291722002173sup001.docx]

**Online Supplement**

**Pre-COVID Brain Functional Connectome Features Prospectively Predict Emergence of Distress Symptoms After Onset of the COVID-19 Pandemic**

**Supplementary Methods**

**Supplementary Tables and Figures**

**Table S1.** Subscale Data of Psychological Assessment

**Table S2.** Eigenvalues and Variance Contribution in Principal Component Analysis

**Table S3.** Identified Links of Distress-Related Functional Connectome

**Table S4.** Node Strength of Distress-Related Functional Connectome

**Figure S1.** Brain Functional Network Parcellation

**Figure S2.** Difference of Distress Symptoms Between Pre- and During-Pandemic

**Figure S3.** Functional Connectivity Matrix without Thresholding

**Figure S4.** Mediation Models in Supplemental Analysis

**Reference**

The supplementary material has been provided by the authors to give readers additional information about their work.

**Supplementary Methods**

1. MRI Protocol Procedure.

We used echo-planar imaging sequence to acquire the rs-fMRI data (TR = 2000 ms, TE = 30 ms, flip angle = 90°, 30 slices, voxel size = 3.75 × 3.75 × 5 mm^3^, field of view = 240 × 240 mm^2^, matrix = 64 × 64, 240 volumes). To achieve high data quality, participants with excessive head motion (transformation distance > 1 mm, rotation angle > 1°) were required to undergo rs-fMRI scanning again until they meet the criteria. High-resolution T1-weighted anatomical MRI images were obtained (TR = 1900 ms, TE = 2.26 ms, flip angle = 9°, 176 slices, voxel size = 1 × 1 × 1mm^3^, matrix size = 256 × 256). During the scanning, participants were instructed to remain relaxed, close their eyes, and stay awake without systematically thinking.

1. Depression Anxiety Stress Scale–21 (DASS-21).

Assessed at T1 (pre-pandemic period: October 13, 2019 to January 19, 2020) and T3 (post-peak period of the pandemic: March 10 to April 18, 2021).

As a popular measure of general distress experienced in the past week, the DASS-21 has 3 dimensions (i.e., depression, anxiety and stress) comprising a general distress construct (Henry and Crawford, 2005; Zanon *et al.*, 2021). Each dimension includes 7 items, and participants are asked to respond to these items from 1 (not at all) to 4 (very much so). The ratings of all items were summed to form a total score, and higher scores indicated higher general distress. Previous studies have suggested that the Chinese version of the DASS-21 shows good reliability and validity among general populations (Wang *et al.*, 2020). In our dataset, the Cronbach's αs of the DASS at T1 and T3 were 0.93 and 0.95 respectively, indicating satisfactory internal reliability.

We employed Kaiser-Meyer-Olkin and Bartlett’s sphericity test to determine whether the data were suitable for PCA, and calculated the average eigenvalue by generating data from three subscales (Li *et al.*, 2020). We followed Kaiser’s rule and discarded components with eigenvalues under 1.0 (Wang et al., 2017), so that we concluded that both waves of the DASS data corresponded to single-factor models (details in online Supplementary Table S2). The single factor of pre-pandemic DASS (eigenvalue = 2.53) accounted for 84.16% of the total variance, and that of during-pandemic DASS (eigenvalue = 2.61) accounted for 87.09%.

1. Impact of Event Scale-Revised (IES-R) and Posttraumatic Stress Disorder Checklist for DSM-5 (PCL-5)

Assessed at T2 (period of community-level pandemic outbreaks: February 1 to April 1, 2020).

The IES-R is a 22-item self-report measure (for DSM-IV) that assesses subjective distress feelings caused by traumatic events (Weiss, 2007). Respondents are asked to identify a specific stressful life event and then indicate how much they were distressed or bothered during the past seven days by each "difficulty" listed (Christianson and Marren, 2012). Items are rated on a 5-point scale ranging from 0 ("not at all") to 4 ("extremely"). The IES-R yields a total score (ranging from 0 to 88) and subscale scores can also be calculated for the intrusion, avoidance, and hyperarousal subscales. In general, the IES-R is not used to diagnosis PTSD, however, cutoff scores for a preliminary diagnosis of PTSD have been cited in the literature (Weiss, 2007).

The PCL-5 is a 20-item self-report checklist of PTSD symptoms based closely on the DSM-5 criteria, including 4 subscales (i.e., intrusion, avoidance, cognition/mood, arousal) (Blevins *et al.*, 2015). Respondents rate each item from 0 ("not at all") to 4 ("extremely") to indicate the degree to which they have been bothered by that particular symptom over the past week. Initial research suggests that a PCL-5 cutoff score between 31-33 is indicative of probable PTSD across samples.

The IES-R and PCL-5 showed satisfactory internal reliability in our dataset (Cronbach's α = 0.97). In the principal component analysis, the single factor (eigenvalue = 5.11) accounted for 72.96% of the total variance when we followed Kaiser’s rule and discarded components with eigenvalues under 1.0, and we attributed it to the CPTS.

**Supplementary Tables and Figures**

**Table S1.** Subscale Data of Psychological Assessment

| Characteristics | | Mean | SD | Range |
| --- | --- | --- | --- | --- |
| **Time 1 (Pre-Pandemic)** | | | | |
| DASS | Depression | 11.12 | 3.40 | 7-19 |
|  | Anxiety | 12.16 | 3.28 | 7-19 |
|  | Stress | 13.70 | 3.56 | 7-21 |
| **Time 2 (During-Pandemic, Community-Level Outbreak)** | | | | |
| IES-R | Intrusion | 12.51 | 4.59 | 8-32 |
|  | Avoidance | 13.13 | 4.77 | 8-31 |
|  | Hyperarousal | 8.91 | 3.39 | 6-22 |
| PCL-5 | Intrusion | 7.44 | 3.03 | 5-19 |
|  | Avoidance | 3.21 | 1.57 | 2-8 |
|  | Cognition/Mood | 10.28 | 3.96 | 7-30 |
|  | Arousal | 8.04 | 3.22 | 6-22 |
| **Time 3 (During-Pandemic, Post-Peak)** | | | | |
| DASS | Depression | 11.38 | 3.71 | 7-21 |
|  | Anxiety | 11.50 | 3.56 | 7-20 |
|  | Stress | 12.86 | 4.22 | 7-21 |

Note. Time 1: October 2019 to January 2020; Time 2: February to April 2020; Time 3: March to April 2021. SD = standard deviation, DASS = Depression Anxiety Stress Scales, IES-R = Impact of Event Scale-Revised, PCL-5 = Posttraumatic Stress Disorder Checklist for DSM-5.

**Table S2.** Eigenvalues and Variance Contribution in Principal Component Analysis

| Assessment | Component | Eigenvalue | % of Variance | % Cumulative |
| --- | --- | --- | --- | --- |
| DASS  (Time 1) ^a^ | **A1** | **2.525*** | **84.16** | 84.16 |
|  | A2 | 0.311 | 10.37 | 94.53 |
|  | A3 | 0.164 | 5.48 | 100.00 |
| IES-R & PCL-5  (Time 2) ^b^ | **B1** | **5.107*** | **72.96** | 72.96 |
|  | B2 | 0.822 | 11.74 | 84.70 |
|  | B3 | 0.315 | 4.51 | 89.21 |
|  | B4 | 0.276 | 3.94 | 93.15 |
|  | B5 | 0.185 | 2.65 | 95.80 |
|  | B6 | 0.164 | 2.35 | 98.15 |
|  | B7 | 0.130 | 1.85 | 100.00 |
| DASS  (Time 3) ^c^ | **C1** | **2.613*** | **87.09** | 87.09 |
|  | C2 | 0.219 | 7.29 | 94.37 |
|  | C3 | 0.169 | 5.63 | 100.00 |

Note. ^a^ Kaiser-Meyer-Olkin (KMO) measure of sampling adequacy = .726, χ2 1 of Bartlett’s sphericity = 199.02 (*p* < .001); ^b^ KMO = .892, χ2 1 = 626.67 (*p* < .001); ^c^ KMO = .761, χ2 1 = 227.32 (*p* < .001). * We kept the components with eigenvalues over 1.0 and highlighted those with boldface. A KMO value over 0.6 and a significance level for the Bartlett's test below 0.05 suggest substantial correlation in the data, which is adequate for factor analysis (Wang *et al.*, 2017).

**Table S3.** Identified Links of Distress-Related Functional Connectome

| Label of Node A | Network Membership of Node A | Label of Node B | Network Membership of Node B | Connection Category | Connection Strength |
| --- | --- | --- | --- | --- | --- |
| L Temporal_1 | DMN | L pCunPCC_1 | DMN | Within | 3.51 |
| L PFC_4 | DMN | L pCunPCC_2 | DMN | Within | 3.57 |
| L Temporal_1 | DMN | R dmPFC_3 | DMN | Within | 3.87 |
| L PFC_5 | DMN | R dmPFC_3 | DMN | Within | 3.80 |
| R Temporal_2 | DMN | R dmPFC_3 | DMN | Within | 3.60 |
| L PFC_4 | DMN | R pCunPCC_2 | DMN | Within | 4.12 |
| L Temporal_1 | DMN | L caudal HIP | DMN | Within | 3.70 |
| L pCunPCC_1 | DMN | L caudal HIP | DMN | Within | 3.62 |
| R Parietal_1 | DMN | L caudal HIP | DMN | Within | 3.76 |
| R dmPFC_1 | DMN | L caudal HIP | DMN | Within | 3.76 |
| R dmPFC_2 | DMN | L caudal HIP | DMN | Within | 4.00 |
| R dmPFC_3 | DMN | L caudal HIP | DMN | Within | 3.92 |
| R pCunPCC_2 | DMN | L caudal HIP | DMN | Within | 3.72 |
| L Temporal_1 | DMN | R caudal HIP | DMN | Within | 3.52 |
| R Parietal_1 | DMN | R caudal HIP | DMN | Within | 3.72 |
| R dmPFC_1 | DMN | R caudal HIP | DMN | Within | 3.59 |
| R dmPFC_2 | DMN | R caudal HIP | DMN | Within | 3.86 |
| R dmPFC_3 | DMN | R caudal HIP | DMN | Within | 3.84 |
| R pCunPCC_2 | DMN | R caudal HIP | DMN | Within | 3.98 |
| L PCC_1 | DAN | R PCC_5 | DAN | Within | 3.52 |
| R dmPFC_1 | DMN | R medial AMYG | AFN | Between | 3.74 |
| R dmPFC_2 | DMN | R medial AMYG | AFN | Between | 3.86 |
| R dmPFC_3 | DMN | R medial AMYG | AFN | Between | 3.62 |
| R dmPFC_3 | DMN | R lateral AMYG | AFN | Between | 3.55 |
| R pCun_1 | CEN | R Temporal_1 | DMN | Between | 3.72 |
| R pCun_1 | CEN | R dmPFC_2 | DMN | Between | 3.52 |
| R pCun_1 | CEN | L caudal HIP | DMN | Between | 3.96 |
| L PCC_5 | DAN | L Parietal_1 | DMN | Between | 3.50 |
| L PCC_3 | DAN | R Parietal_1 | DMN | Between | 3.57 |
| L PCC_5 | DAN | R Parietal_1 | DMN | Between | 3.50 |
| R PCC_4 | DAN | R Parietal_1 | DMN | Between | 4.01 |
| R PCC_5 | DAN | R Parietal_1 | DMN | Between | 3.79 |
| L PCC_6 | DAN | R dmPFC_3 | DMN | Between | 3.68 |
| R PCC_5 | DAN | R dmPFC_3 | DMN | Between | 3.88 |
| L PCC_1 | DAN | R pCunPCC_2 | DMN | Between | 3.90 |
| R FEF_1 | DAN | R pCunPCC_2 | DMN | Between | 3.54 |
| L PCC_1 | DAN | L caudal HIP | DMN | Between | 3.83 |
| L PCC_5 | DAN | L caudal HIP | DMN | Between | 4.03 |
| R PCC_1 | DAN | L caudal HIP | DMN | Between | 3.84 |
| L PCC_1 | DAN | R caudal HIP | DMN | Between | 3.85 |
| L OFC_1 | AFN | R dmPFC_1 | DMN | Between | 3.50 |
| L Temporal pole_1 | AFN | R dmPFC_1 | DMN | Between | 3.58 |
| R Temporal pole_1 | AFN | R dmPFC_1 | DMN | Between | 3.70 |
| L OFC_1 | AFN | R dmPFC_3 | DMN | Between | 3.60 |
| L Temporal pole_1 | AFN | R dmPFC_3 | DMN | Between | 3.87 |
| L Temporal pole_2 | AFN | R dmPFC_3 | DMN | Between | 3.69 |
| R Temporal pole_1 | AFN | R dmPFC_3 | DMN | Between | 3.52 |
| L Temporal pole_1 | AFN | R pCunPCC_2 | DMN | Between | 3.69 |
| L Temporal pole_2 | AFN | R pCunPCC_2 | DMN | Between | 3.60 |
| R OFC_1 | AFN | L caudal HIP | DMN | Between | 3.69 |
| R Temporal pole_1 | AFN | L caudal HIP | DMN | Between | 3.56 |
| R medial AMYG | AFN | L caudal HIP | DMN | Between | 3.78 |
| R lateral AMYG | AFN | L caudal HIP | DMN | Between | 3.54 |
| L Somatomotor_6 | SMN | R dmPFC_3 | DMN | Between | 4.07 |
| L mPFC_2 | VAN | R Parietal_1 | DMN | Between | 3.54 |
| R mPFC_1 | VAN | R dmPFC_1 | DMN | Between | 3.76 |
| L mPFC_2 | VAN | R dmPFC_3 | DMN | Between | 4.17 |
| L Visual_1 | VN | R Parietal_1 | DMN | Between | 3.88 |
| L Visual_7 | VN | L caudal HIP | DMN | Between | 4.05 |
| R Visual_3 | VN | L caudal HIP | DMN | Between | 3.57 |
| R Visual_3 | VN | R caudal HIP | DMN | Between | 3.71 |
| L Parietal_1 | DMN | R PCC_1 | DAN | Between | 3.64 |
| L Parietal_1 | DMN | R PCC_5 | DAN | Between | 4.00 |
| L PCC_1 | DAN | R pCun_1 | CEN | Between | 3.52 |
| L PCC_5 | DAN | R pCun_1 | CEN | Between | 3.54 |
| L Temporal pole_1 | AFN | R pCun_1 | CEN | Between | 3.54 |
| L Temporal pole_2 | AFN | R pCun_1 | CEN | Between | 3.66 |
| R Temporal pole_1 | AFN | R pCun_1 | CEN | Between | 3.62 |
| L Visual_7 | VN | R pCun_1 | CEN | Between | 3.55 |
| R Visual_3 | VN | R pCun_1 | CEN | Between | 3.89 |

Note. The information of node label and network membership was obtained from the 100-brain-area parcellation and Human Brainnetome atlas (Fan *et al.*, 2016; Schaefer *et al.*, 2018). For location information of seed regions, see Table S2. L = left, R = right, DMN = default mode network, CEN = central executive network, DAN = dorsal attention network, AFN = cortical affective network, VN = visual network, VAN = ventral attention network, AMYG = amygdala, HIP = hippocampus, dmPFC = dorsomedial prefrontal cortex, pCun = precuneus, PCC = posterior cingulate cortex, OFC = orbitofrontal cortex, FEF = frontal eye field.

**Table S4.** Node Strength of Distress-related Functional Connectome

| Node Label | Network Membership | MNI Coordinate | | | Node Strength | Number of Links |
| --- | --- | --- | --- | --- | --- | --- |
|  |  | x | y | z |  |  |
| L caudal HIP | DMN | -20 | -40 | 3 | 63.08 | 17 |
| R dmPFC_3 | DMN | 26 | 24 | 50 | 56.26 | 15 |
| R_pCun_1 | CEN | 9 | -66 | 43 | 37.76 | 10 |
| R_Par_1 | DMN | 55 | -51 | 31 | 30.92 | 8 |
| R caudal HIP | DMN | 30 | -33 | -8 | 29.70 | 8 |
| R_pCunPCC_2 | DMN | 7 | -52 | 31 | 26.54 | 7 |
| R dmPFC_1 | DMN | 7 | 48 | 1 | 25.78 | 7 |
| L_PCC_1 | DAN | -47 | -58 | -13 | 18.42 | 5 |
| L Temporal pole_1 | AFN | -32 | 2 | -37 | 15.44 | 4 |
| R dmPFC_2 | DMN | 11 | 50 | 39 | 15.10 | 4 |
| R_PCC_5 | DAN | 14 | -52 | 66 | 14.96 | 4 |
| L_PCC_5 | DAN | -6 | -60 | 56 | 14.79 | 4 |
| R medial AMYG | AFN | 24 | 1 | -20 | 14.72 | 4 |
| R Temporal pole_1 | AFN | 38 | 1 | -38 | 14.68 | 4 |
| L Temporal_1 | DMN | -55 | -4 | -20 | 14.59 | 4 |
| L Temporal pole_2 | AFN | -57 | -33 | -21 | 11.21 | 3 |
| R Visual_3 | VN | 49 | -60 | -11 | 11.10 | 3 |
| L Par_1 | DMN | -57 | -50 | 12 | 10.74 | 3 |
| L OFC_1 | AFN | -14 | 32 | -20 | 7.72 | 2 |
| L PFC_4 | DMN | -24 | 61 | -1 | 7.69 | 2 |
| L mPFC_2 | VAN | -11 | -34 | 45 | 7.53 | 2 |
| R lateral AMYG | AFN | 27 | 0 | -25 | 7.47 | 2 |
| L Visual_7 | VN | -47 | -71 | 11 | 7.29 | 2 |
| L pCunPCC_1 | DMN | -11 | -56 | 13 | 7.13 | 2 |
| R PCC_1 | DAN | 50 | -62 | 16 | 7.05 | 2 |
| R FEF_1 | DAN | 28 | -3 | 59 | 4.00 | 1 |
| R PCC_4 | DAN | 27 | -67 | 51 | 3.88 | 1 |
| L PFC_5 | DMN | -9 | 48 | 41 | 3.80 | 1 |
| L PCC_3 | DAN | -24 | -68 | 49 | 3.76 | 1 |
| R OFC_1 | AFN | 12 | 35 | -20 | 3.70 | 1 |
| R Temporal_1 | DMN | 62 | -23 | -19 | 3.62 | 1 |
| L Visual_1 | VN | -26 | -34 | -17 | 3.62 | 1 |
| R Temporal_2 | DMN | 51 | 7 | -18 | 3.60 | 1 |
| L pCunPCC_2 | DMN | -6 | -53 | 33 | 3.57 | 1 |
| L PCC_6 | DAN | -22 | -51 | 66 | 3.57 | 1 |
| R mPFC_1 | VAN | 11 | -31 | 45 | 3.50 | 1 |
| L Somatomotor_6 | SMN | -6 | -29 | 70 | 3.50 | 1 |

Note. The node strength was calculated by summing the *t* value of the corresponding connections that represented the correlates between functional connectome and difference in distress (all the selected *t* values are positive). The node information was extracted from the 100-brain-area parcellation and Human Brainnetome atlas (Fan *et al.*, 2016; Schaefer *et al.*, 2018). L = left, R = right, DMN = default mode network, CEN = central executive network, DAN = dorsal attention network, AFN = cortical affective network, VN = visual network, VAN = ventral attention network, AMYG = amygdala, HIP = hippocampus, dmPFC = dorsomedial prefrontal cortex, pCun = precuneus, PCC = posterior cingulate cortex, OFC = orbitofrontal cortex, FEF = frontal eye field.

**
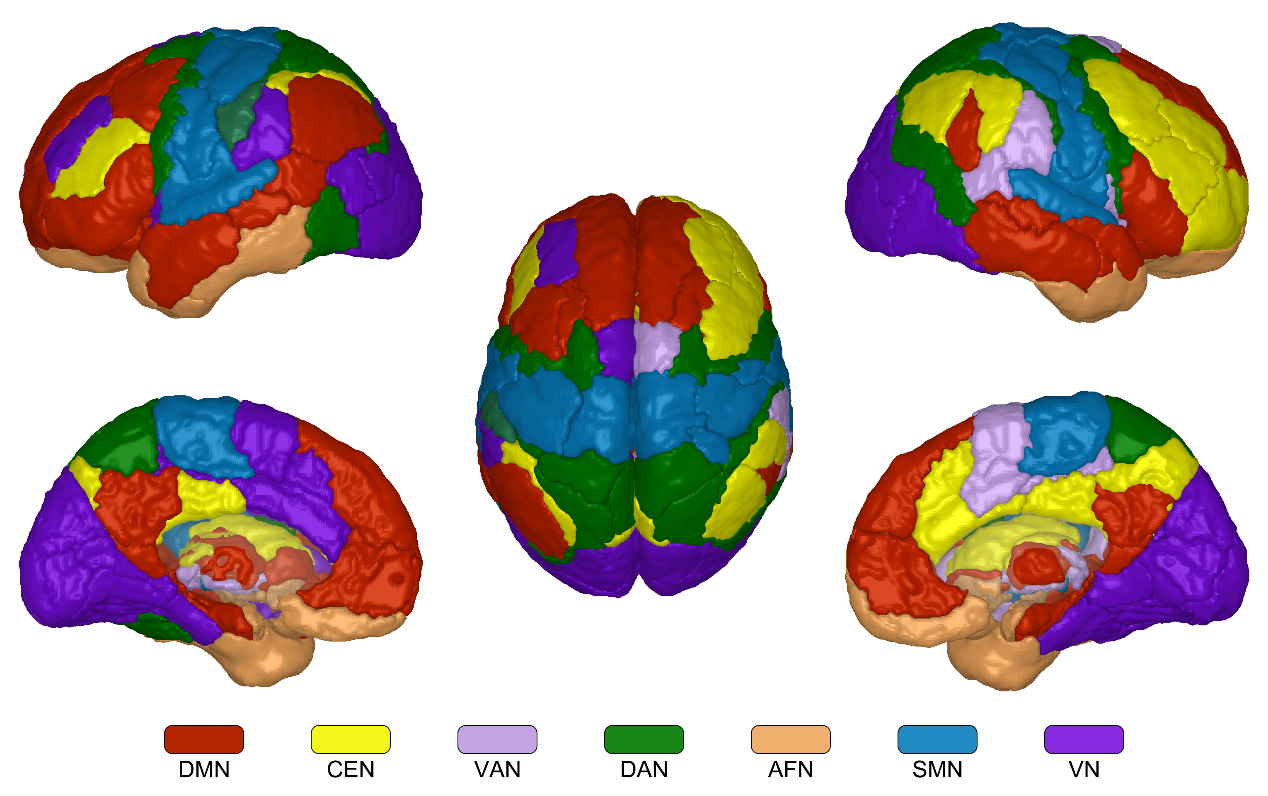
**

**Figure S1.** Brain functional network parcellation. The cortical and subcortical brain areas were parceled with 136 seed regions, and these seed regions were assigned to 7 macroscale networks in accordance with priori hypothesis: the default mode network (DMN), central executive network (CEN), ventral attention network (VAN), dorsal attention network (DAN), cortical affective network (AFN), sensorimotor network (SMN) and visual network (VN). Brain atlas and corresponding information available online at https://osf.io/fkdtx/files/.


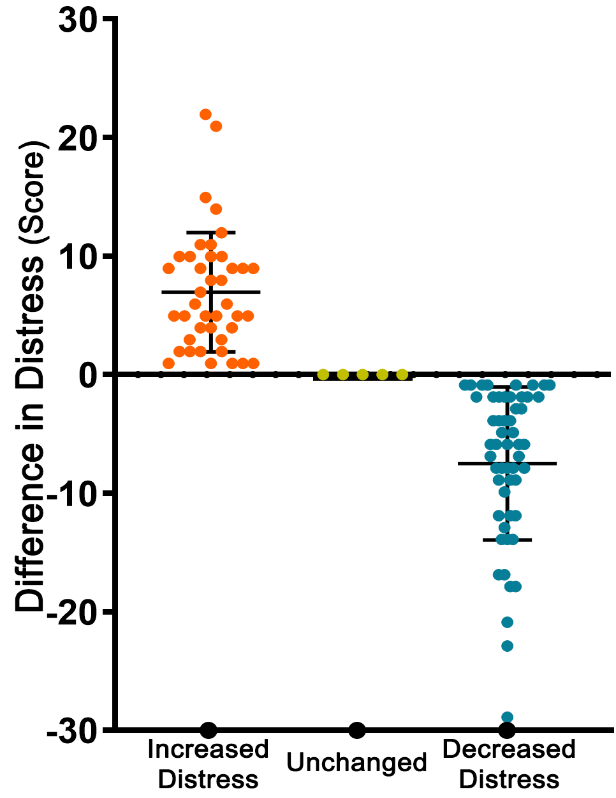


**Figure S2.** Difference of distress symptoms between pre- and during-pandemic (distress score of during-pandemic – pre-pandemic). Individuals were assigned to three groups as increased distress, unchanged, and decreased distress group based on the differences between the time period.

**
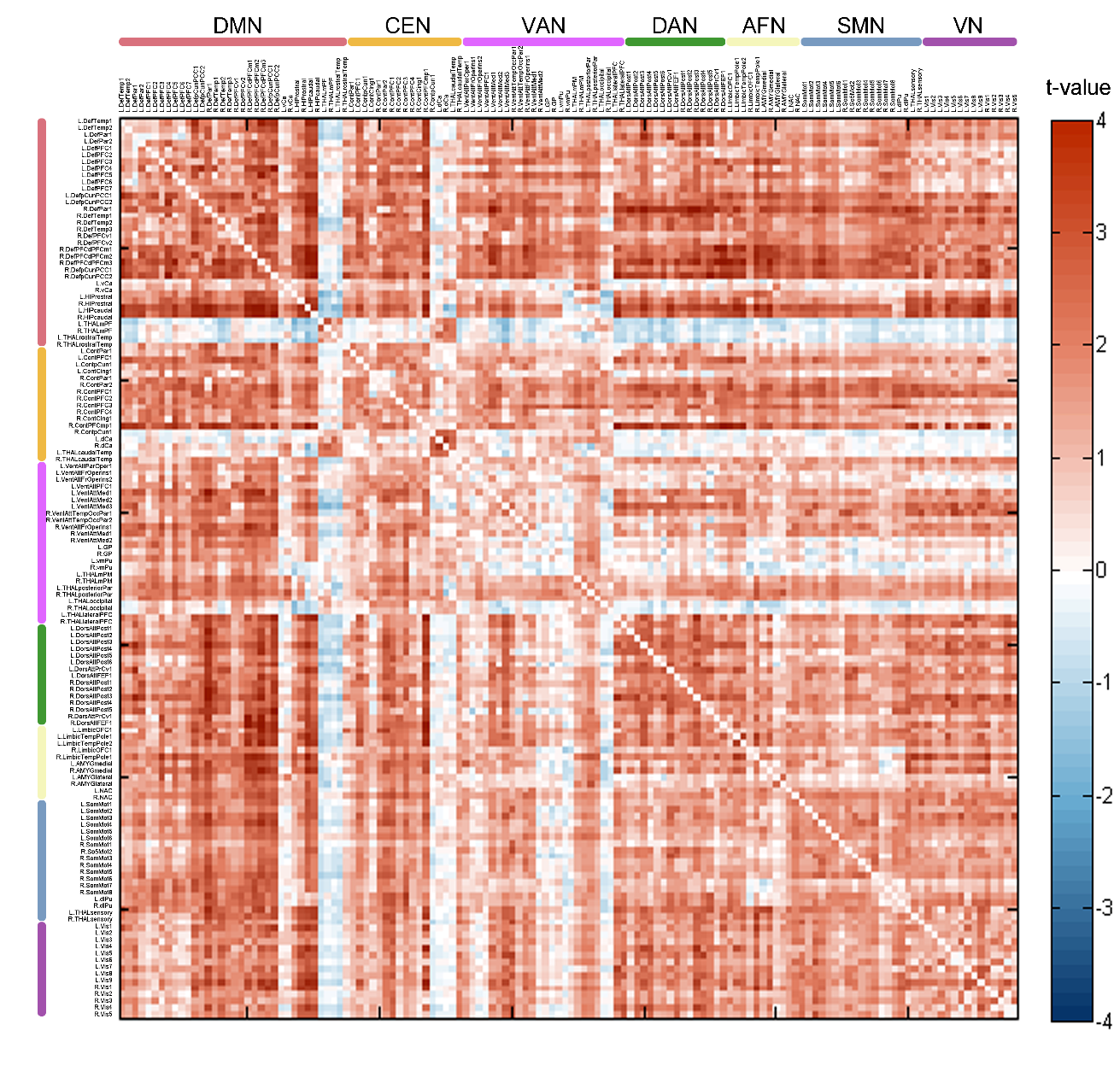
**

**Figure S3.** Functional connectivity matrix without thresholding. The weighted matrix represents the correlation of specific connections to difference in distress, and the rows and columns represent 136 seed regions with network membership. DMN = default mode network, CEN = central executive network, DAN = dorsal attention network, AFN = cortical affective network, VN = visual network, VAN = ventral attention network.


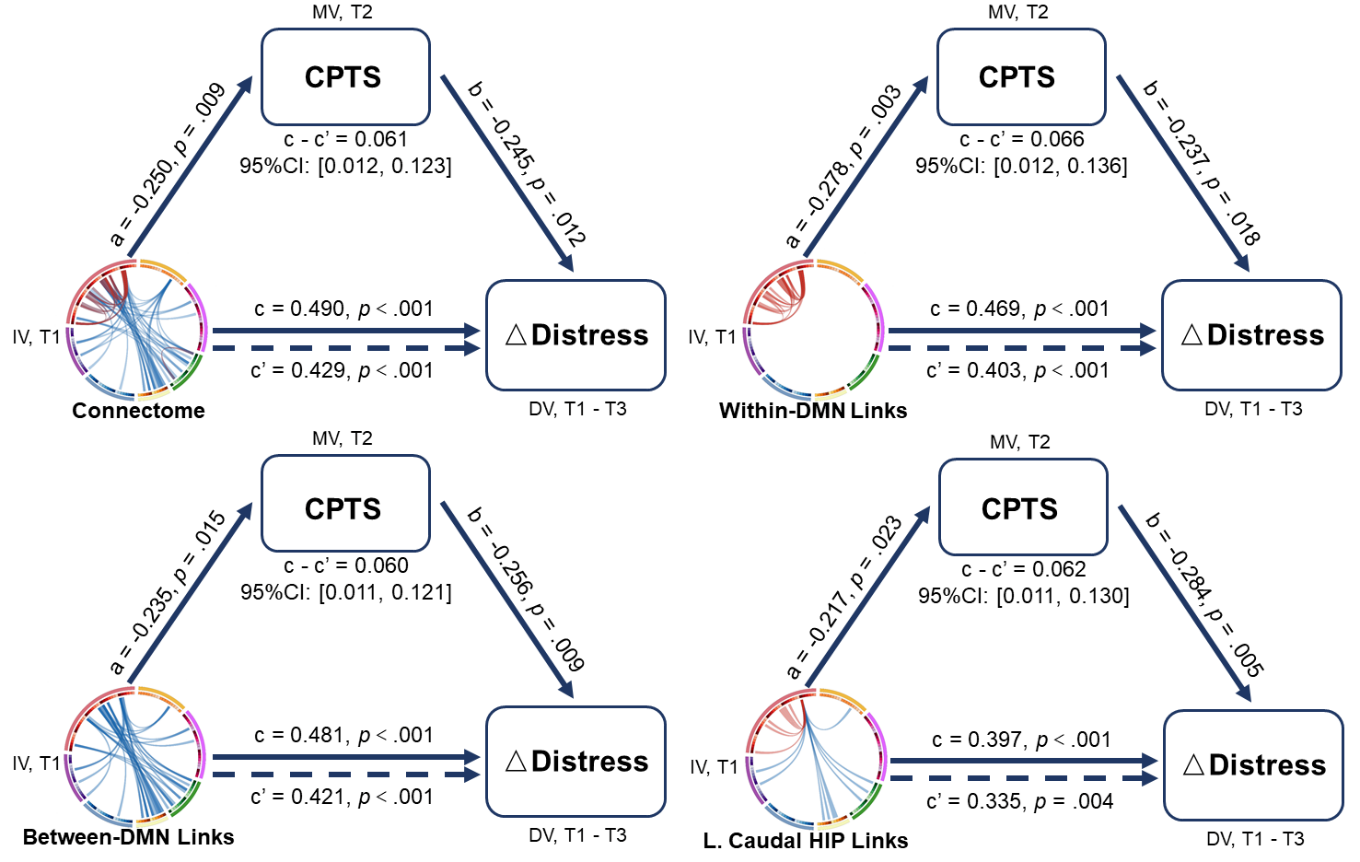


**Figure S4.** Mediation models in supplemental analysis. COVID-19 posttraumatic stress (CPTS) underlies the correlates between brain features and distress alterations. The indirect effect of CPTS (c - c’) is significant among the four models. Age, sex, head motion, and scores of the self-rating life events checklist and socio-economic status scale were regarded as covariates in the mediation analyses, and the coefficients in pathways (a, b, c, and c’) were exhibited as standard regression coefficients. DMN = default mode network, L. = left, HIP = hippocampus, △Distress = pre-pandemic – during-pandemic distress score.

**Supplementary Reference**

**Blevins CA, Weathers FW, Davis MT, Witte TK, Domino JL** (2015) The Posttraumatic Stress Disorder Checklist for DSM-5 (PCL-5): Development and Initial Psychometric Evaluation. United States, United States *Journal of traumatic stress* **28**, 489–498.

**Christianson S, Marren J** (2012) The Impact of Event Scale - Revised (IES-R). United States *Medsurg nursing : official journal of the Academy of Medical-Surgical Nurses* **21**, 321–322.

**Fan L, Li H, Zhuo J, Zhang Y, Wang J, Chen L, Yang Z, Chu C, Xie S, Laird AR, Fox PT, Eickhoff SB, Yu C, Jiang T** (2016) The Human Brainnetome Atlas: A New Brain Atlas Based on Connectional Architecture. *Cerebral Cortex* **26**, 3508–3526.

**Henry JD, Crawford JR** (2005) The short-form version of the Depression anxiety stress scales (DASS-21): Construct validity and normative data in a large non-clinical sample. *British Journal of Clinical Psychology* **44**, 227–239.

**Li Y, Zhuang K, Yi Z, Wei D, Sun J, Qiu J** (2020) The trait and state negative affect can be separately predicted by stable and variable resting-state functional connectivity. *Psychological Medicine* 1–11.

**Schaefer A, Kong R, Gordon EM, Laumann TO, Zuo X-N, Holmes AJ, Eickhoff SB, Yeo BTT** (2018) Local-Global Parcellation of the Human Cerebral Cortex from Intrinsic Functional Connectivity MRI. *Cerebral Cortex* **28**, 3095–3114.

**Wang C, Pan R, Wan X, Tan Y, Xu L, McIntyre RS, Choo FN, Tran B, Ho R, Sharma VK, Ho C** (2020) A longitudinal study on the mental health of general population during the COVID-19 epidemic in China. Elsevier *Brain, Behavior, and Immunity* **87**, 40–48.

**Wang F, Peng K, Chechlacz M, Humphreys GW, Sui J** (2017) The Neural Basis of Independence Versus Interdependence Orientations: A Voxel-Based Morphometric Analysis of Brain Volume. *Psychological Science* **28**, 519–529.

**Weiss DS** (2007) The Impact of Event Scale: Revised. Springer US *Cross-Cultural Assessment of Psychological Trauma and PTSD* 219–238.

**Zanon C, Brenner RE, Baptista MN, Vogel DL, Rubin M, Al-Darmaki FR, Gonçalves M, Heath PJ, Liao HY, Mackenzie CS, Topkaya N, Wade NG, Zlati A** (2021) Examining the Dimensionality, Reliability, and Invariance of the Depression, Anxiety, and Stress Scale–21 (DASS-21) Across Eight Countries. *Assessment* **28**, 1531–1544.
